# Supplementary material for: MetaRibo-Seq measures translation in microbiomes
Source: Nat Commun. 2020 Jun 29;11:3268. doi: 10.1038/s41467-020-17081-z (PMC7324362; doi:10.1038/s41467-020-17081-z)
Supplement: Supplementary file 10 — Supplementary Data 7 [file 41467_2020_17081_MOESM10_ESM.zip › File2/Confidence_High_Taxonomy/410604_out.krona.html]

Javascript must be enabled to view this page.

members
magnitude
magnitudeUnassigned
count
unassigned
taxon
rank

410604\_out

18

superkingdom
18
2

1224
phylum
18

18
class
28216

80840
18
order

995019
family
18

genus
18
40544


SRS015264\_contig\_number\_21209SRS015663\_contig\_number\_19267SRS015782\_contig\_number\_4775SRS044535\_contig\_number\_contig-100\_296.147227SRS047044\_contig\_number\_13251SRS050026\_contig\_number\_2293SRS051031\_contig\_number\_34953SRS055017\_contig\_number\_27662SRS077086\_contig\_number\_contig-100\_414.86095SRS098571\_contig\_number\_5999SRS104036\_contig\_number\_contig-100\_350.62344SRS104327\_contig\_number\_contig-100\_420.50839SRS105082\_contig\_number\_20250SRS142923\_contig\_number\_15732SRS143876\_contig\_number\_32378SRS145497\_contig\_number\_14103SRS146764\_contig\_number\_33087SRS148253\_contig\_number\_contig-100\_420.96828
40545
18
species
